# Telomere length in cervical exfoliated cells, interaction with HPV genotype, and cervical cancer occurrence among high‐risk HPV‐positive women

**DOI:** 10.1002/cam4.2246

**Published:** 2019-06-26

**Authors:** Xiaojun Chen, Sun Wei, Hongxia Ma, Guangfu Jin, Zhibin Hu, Han Suping, Dake Li, Dong Hang, Xiaohua Wu, Ni Li

**Affiliations:** ^1^ Department of Gynecologic Oncology Fudan University Shanghai Cancer Center Shanghai China; ^2^ Department of Gynecology The First Affiliated Hospital of Nanjing Medical University Nanjing China; ^3^ Department of Epidemiology and Biostatistics, School of Public Health Nanjing Medical University Nanjing China; ^4^ Department of Gynecologic Oncology Nanjing Maternity and Child Health Hospital Nanjing China; ^5^ Jiangsu Key Lab of Cancer Biomarkers, Prevention and Treatment, Collaborative Innovation Center for Cancer Personalized Medicine Nanjing Medical University Nanjing China; ^6^ National Office for Cancer Prevention and Control Cancer Institute and Hospital, Chinese Academy of Medical Sciences Beijing China

**Keywords:** biomarker, cervical cancer, human papillomavirus, telomere

## Abstract

**Background:**

Although high‐risk human papillomavirus (HR‐HPV) infection is recognized as the main cause of cervical cancer, only a minority of HPV‐infected women develop this malignancy. Increasing evidence suggests that alterations of telomere length might be implicated in carcinogenesis. However, the association between cervical cancer and telomere length remains unknown.

**Methods:**

This case‐control study included 591 cervical cancer patients and 373 cancer‐free controls, all of whom were infected with HR‐HPV. Relative telomere length (RTL) in cervical cancer exfoliated cells was measured by quantitative PCR. Odds ratios (ORs) and 95% confidence intervals (CIs) were calculated by logistic regression analysis.

**Results:**

HPV16, 18, 52, and 58 were common in both case and control groups. The proportion of HPV16 infection tended to increase across the quartiles of RTL (*P*_trend_ < 0.001). There was no statistically significant association of RTL with tumor differentiation, histological type, and FIGO stage. After adjustment for age and HPV types, the lowest quartile of RTL presented a 49% lower risk (OR = 0.51, 95% CI: 0.35, 0.76; *P* < 0.001) than those with the highest quartile of RTL. There was also a dose‐response relationship of shorter RTL on lower risk of cervical cancer (*P*_trend_ < 0.001).

**Conclusion:**

Shortened telomere length in cervical exfoliated cells was related to the lower risk of cervical cancer among HR‐HPV‐positive women, which might help to improve cervical cancer screening and surveillance. Further prospective studies with large sample should be designed to validate our preliminary findings, and evaluate the potential efficacy of telomere length for cervical cancer screening.

## Abstract 2

New triage biomarkers for human papillomavirus‐positive women are necessary to improve the identification of individuals at high risk of cervical cancer progression. Shorter telomere length in cervical exfoliated cells was associated with the lower risk of cervical cancer. Shortened telomere length may act as a protective factor in cervical carcinogenesis and represent a potential biomarker for this malignancy.

## 1 INTRODUCTION

Cervical cancer is one of the most common malignancies in women worldwide.^1^ Infection of high‐risk human papillomavirus (HR‐HPV) has been defined as the necessary cause of the disease.^2^ At least 13 high‐risk types have been identified, including HPV16, 18, 31, 33, 35, 39, 45, 51, 52, 56, 58, 59, and 68. HPV16 and 18 are the most frequent types in both cases and controls worldwide, while HPV52 and 58 are the most frequent types in both cases and controls in China. Our previous study also found that HPV 16, 18, 52, and 58 were the most common types causing cervical cancer among Chinese women.^3^, ^4^, ^5^, ^6^ Only a minority of HR‐HPV infections become persistent and finally produces cancerous lesions, suggesting other factors (eg, host immunity and genetic susceptibility) might affect the carcinogenesis.^7^ It might take 10‐15 years from the initial HR‐HPV infection to cancer appearance,^8^ thus allowing sufficient time for potential cervical screening. As a secondary prevention effort, early screening of cervical cancer and precursor lesions could reduce the incidence and mortality of cervical cancer.^9^ The current screening methods are primarily based on cytologic diagnosis and HPV testing. However, cytology triage is limited by relatively low sensitivity and high dependence on a cytologist's experience, and HPV testing has poor positive predictive value (less than 50%) for high‐grade lesions, since HPV infection is a frequent event and nearly 90% of infections resolve spontaneously within 2 years.^10^, ^11^, ^12^ Therefore, the exploration of new triage biomarkers for HPV‐positive women is necessary to improve the identification of high‐risk individuals for cervical cancer.

HR‐HPV causes cervical cancer by increasing E6 and E7 oncogene expression that inactivate the tumor suppressor pRb and p53.^13^ More studies reported that HR‐HPV E7and E6 could regulate hTERT expression and induce telomerase activation, which elongates telomere length and contributes to cellular immortalization and tumorigenesis.^14^, ^15^ Telomeres are repetitive nucleotide sequences(TTAGGG) at the chromosome end, and play a critical role in maintaining chromosomal integrity and stability through prohibiting abnormal events.^16^ During somatic cell divisions, progressive shortening of telomeres occurs due to the incomplete replication at 3′ end of chromosomes. In addition, telomeres may shorten as a result of aging, chronic inflammation, cigarette smoking, and oxidative stress.^17^, ^18^, ^19^ Extremely short and dysfunctional telomeres induce chromosomal damage, which might trigger cellular senescence and/or apoptosis.^20^ In contrast, the telomerase reactivation could keep stabilization of telomere length, which might result in continuous cell divisions, a hallmark of advanced malignancies.^21^

Numerous epidemiologic studies evaluated the association of cancer risk and telomere length in tissues or peripheral blood leukocytes, but the conclusion was conflicting. In a recent meta‐analysis of prospective studies, the marginally positive association was confirmed between longer telomere length in peripheral blood leukocytes and overall cancer risk, and the association was stronger for lung cancer.^22^ A recent Mendelian randomization study also reported increased telomere length was associated with higher risk for various cancers, including glioma, ovarian cancer, lung adenocarcinoma, endometrial cancer, and so on.^23^ These studies suggested that telomeres might play critical roles in different cancers. There were limited studies for cervical cancer.

To address the paucity, this study intended to evaluate the association between telomere length and cervical cancer risk by determining telomere length in cervical exfoliated cells from 591 cervical cancer patients and 373 cancer‐free controls within a large‐scale cervical screening program. The participants in the current study were all HR‐HPV positive, allowing us to explore the potential interaction between HR‐HPV type and telomere length.

## 2 MATERIALS AND METHODS

### 2.1 Study participants

We recruited participants from a large‐scale cervical screening program who attended gynecological examination in Cancer Institute and Hospital, Chinese Academy of Medical Sciences during January 2010 and July 2012. Cases were histologically confirmed invasive cervical cancer. We excluded those who had recurrent cervical cancer or a history of other malignancies, and those who undergone chemo‐radio therapy before sampling. HR‐HPV‐positive controls were free of liquid‐based cytological abnormalities and were frequency‐matched to the cancer cases by age (±5 years). Cervical exfoliated cells were collected by gynecologists using special soft brushes. Exfoliated cells were used for HPV and Relative telomere length (RTL) tests.

Finally, there were 373 controls and 591 cases included in our study. Written informed consent was obtained, and this study was approved by the ethics committees of Nanjing Medical University and Chinese Academy of Medical Sciences.

### 2.2 HPV genotyping

Detailed procedure for HPV genotyping was described previously.^24^ In brief, genomic DNA of cervical cell specimens was extracted manually by QIAamp DNA Mini Kit (Qiagen, Valencia, CA, USA). The DNA quality was assessed by PCR with the housekeeping gene β‐actin (forward primer, 5′‐GAAATCGTGCGTGACATTAA‐3′; reverse primer 5′‐AAGGAAGGCTGGAAGAGTG‐3′). All β‐actin positive specimens were introduced for HPV DNA tests by a HPV GenoArray Test Kit (HybriBio, Beijing, China), the Chinese FDA‐approved assay for HPV genotyping, which could identify 21 HPV types simultaneously (13 high‐risk types: HPV16, 18, 31, 33, 35, 39, 45, 51, 52, 56, 58, 59, and 68; two intermediate‐risk types 53 and 66; and six low‐risk HPV types: HPV6, 11, 42, 43, 44, and 81).

### 2.3 Measurement of RTL

Relative telomere length was determined by quantitative PCR.^25^ Briefly, RTL was calculated by the ratio of telomere repeat copy number (T) to single gene copy number (S). DNA from five controls were equally pooled as the reference, then serially diluted 1:2 (ranging from 8 to 0.25 ng/μL) to establish the standard curve for assessing the interplate variation in PCR. The coefficient of determination (*R^2^*) was 0.99 or higher for each reaction. The primers for single‐copy gene (36B4) were 36B4u (5′‐CAGCAAGTGGGAAGGTGTAATCC‐3′) and 36B4d (5′‐CCCATTCTATCATCAACGGGTACAA‐3′). The PCR primers for telomere were TEL1 (5′‐GGTTTTTGA[GGGTGA]_4_GGGT‐3′) and TEL2 (5′‐TCCCGACTAT[CCCTAT]_4_CCCTA‐3′). Each well contained 10 μL SYBR^®^ Green PCR Master Mix with a final DNA concentration of 5 ng/μL. The assay was performed in a 384‐well plate using 7900HT Real‐Time PCR System (Applied Biosystems, CA). Technicians were blinded to the study design and case‐control status. All samples were analyzed in duplicate and the mean of their results was used for statistical analysis. RTL was calculated by Cawthon's formula 2^−ΔΔCt^.^26^ Quality control samples were interspersed throughout assays, and the average interplate and the intraplate variations were 10.2% and 8.7%, respectively.

### 2.4 Statistical analysis

Pearson χ^2^ test was introduced to compare categorical variables between cases and controls, such as sex, age, and HPV types. Unconditional logistic regression was applied to assess the odds ratio (OR) and 95% confidence interval (CI), with adjustment for HPV type and age. *P* for trend was calculated by treating RTL categories as ordinal predictors in multivariate regression models. Statistical significance of interaction was assessed by using the Wald test for cross‐product terms of RTL and HPV types in logistic regression models. Stata version 9.2 (Stata Corp. LP) was used for all statistical analysis.

## 3 RESULTS

Demographic and clinical characteristics of participants were shown in Table 1. HPV16, 18, 52, and 58 were common for both cancer cases and controls. There were no statistically significant differences between two groups on age, HPV18, and multiple infections (all *P* > 0.05); Patients with cervical cancer had a higher proportion of HPV16 infection (*P* < 0.001) and lower proportions of HPV52 and HPV58 (both *P* < 0.05) than healthy controls. Most of the cases were squamous carcinoma (94.9%) and at FIGO I/II stage (83.8%).

**Table 1:** Characteristics of cervical cancer patients and cancer‐free controls

Variable	Cases (N = 591)	Controls (N = 373)	*P* a
	n (%)	n (%)	
Age (years)			0.32
<50	339 (57.4)	226 (60.6)	
≥50	252 (42.6)	147 (39.4)	
HPV16			<0.001
Negative	268 (45.3)	300 (80.4)	
Positive	323 (54.7)	73 (19.6)	
HPV18			0.39
Negative	532 (90.0)	342 (91.7)	
Positive	59 (10.0)	31 (8.3)	
HPV52			<0.001
Negative	488 (82.6)	272 (72.9)	
Positive	103 (17.4)	101 (27.1)	
HPV58			<0.01
Negative	536 (90.7)	315 (84.4)	
Positive	55 (9.3)	58 (15.6)	
Others^b^			<0.001
Negative	426 (72.1)	211 (56.6)	
Positive	165 (27.9)	162 (43.4)	
Multiple infection			0.26
No	486 (82.2)	317 (85.0)	
Yes	105 (17.8)	56 (15.0)	
Grade^c^			
High	27 (8.0)		
Middle	178 (52.7)		
Low	133 (39.3)		
Histology^d^			
Squamous	536 (94.9)		
Adeno/adenosquamous	29 (5.1)		
FIGO stage^e^			
I/II	366 (83.8)		
III/IV	71 (16.2)		

Abbreviations: FIGO, International Federation of Gynecology and Obstetrics; HPV, human papillomavirus; RTL, relative telomere length.

a The Pearson χ^2^ test for proportions and the Wilcoxon rank‐sum test for relative telomere length.

b Others included HPV31, 33, 35, 39, 45, 51, 56, 59, 66, and 68.

c Grade information was available in 338 cervical cancer cases. Low, intermediate, and high were correspond to low‐grade (G1, high cell differentiation), intermediate‐grade (G2, moderate cell differentiation), and high‐grade (G3, poor cell differentiation) squamous cell cervical cancer, respectively (WHO,2014).

d Histological information was available in 565 cases. Histology types were defined to squamous and adeno/adenosquamous cell cervical cancer (WHO, 2014).

e FIGO stage information was available for 437 cervical cancer cases. FIGO stage was based on FIGO 2007.

We first analyzed the association between the features of cervical cancer and RTL (Table 2). We found that the proportion of HPV16 infection tended to increase across the quartiles of RTL (*P*_trend_ < 0.001). There was no statistically significant association of RTL with tumor differentiation, histological type, and FIGO stage.

**Table 2:** Association between the characteristics of cervical cancer patients and relative telomere length

Variable	Relative telomere length				*P* a
	Quartile 1 (n = 148)	Quartile 2 (n = 148)	Quartile 3 (n = 147)	Quartile 4 (n = 148)	
Age, mean (SD)	48.4 (8.05)	49.3 (7.71)	48.4 (8.65)	49.2 (8.75)	0.38
HPV16, n (%)	64 (43.24)	71 (47.97)	82 (55.78)	106 (71.62)	<0.001
HPV18, n (%)	20 (13.51)	10 (6.76)	20 (13.61)	9 (6.08)	0.22
HPV52, n (%)	29 (19.59)	29 (19.59)	24 (16.33)	21 (14.19)	0.22
HPV58, n (%)	16 (10.81)	12 (8.11)	14 (9.52)	13 (8.78)	0.70
Others, n (%)	49 (33.11)	51 (34.46)	32 (21.77)	33 (22.30)	0.02
Multiple infections, n (%)	28 (18.92)	24 (16.22)	24 (16.33)	29 (19.59)	0.89
High/middle grade, n (%)	57 (58.76)	51 (60.71)	49 (62.03)	48 (61.54)	0.71
Squamous histology, n (%)	140 (95.89)	134 (95.04)	129 (93.48)	133 (95.00)	0.65
FIGO I/II stage, n (%)	85 (78.70)	96 (82.76)	92 (91.09)	93 (83.04)	0.26

Abbreviations: FIGO, International Federation of Gynecology and Obstetrics; HPV, human papillomavirus; SD, standard deviation.

a The ANOVA test for age and the Cochran‐Armitage test for proportions.

In Table 3, RTL and cervical cancer presented the positive association. Compared with patients with the highest quartile of RTL, participants with the lowest quartile demonstrated the 49% lower risk (OR = 0.51, 95% CI: 0.35, 0.76; *P* < 0.001). The dose‐response relationship was observed between shorter RTL and lower risk of cervical cancer (*P*_trend_ < 0.001).

**Table 3:** Association between relative telomere length and cervical cancer risk

Relative telomere length	Cases	Controls	OR (95% CI)^b^		*P* b	*P*_trend_
	n (%)	n (%)				
Quartile^a^	≥1.58	203 (34.35)	93 (24.93)	1.00 (Ref)		<0.001
	1.13‐1.58	121 (21.15)	93 (24.93)	0.56 (0.38,0.83)	0.003	
	0.84‐1.13	115 (18.78)	94 (25.21)	0.47 (0.32,0.70)	<0.001	
	<0.84	152 (25.72)	93 (24.93)	0.51 (0.35,0.76)	<0.001	

Abbreviations: CI, confidential interval; OR, odds ratio; Ref, reference.

a According to the quartile distribution of relative telomere length among controls.

b Logistic regression models with adjustment for age, HPV16, 18, 52, 58, and the other high risk types. *P*_trend _was calculated by treating quartiles as ordinal predictors in multivariate regression models.

We further performed stratified analysis according dosage and HPV type (Table 4). The protective dose‐response relationship remained statistically significant in subgroups of age <50, HPV16 negative, HPV18 negative, HPV52 negative and positive, HPV58 negative, other type positive, and multiple type positive and negative. We also found a potential interaction between RTL and HPV16 (*P* = 0.04), suggesting a lower cancer risk for those with short RTL and non‐HPV16 infection than other participants.

**Table 4:** Stratified analysis of the association between relative telomere length and cervical cancer risk

Variable	Relative telomere length, OR (95% CI)^a^				*P*_trend_	*P*_interaction_
	Quartile 4	Quartile 3	Quartile 2	Quartile 1		
Age (years)						
<50	1.00 (Ref)	0.59 (0.35, 1.01)	0.42 (0.25, 0.70)	0.39 (0.23, 0.66)	<0.001	0.44
≥50	1.00 (Ref)	0.55 (0.31, 0.98)	0.50 (0.26, 0.95)	0.72 (0.39, 1.33)	0.18	
HPV16						
Negative	1.00 (Ref)	0.53 (0.35, 0.84)	0.40 (0.25, 0.65)	0.40 (0.25, 0.65)	<0.001	0.04
Positive	1.00 (Ref)	0.76 (0.25, 1.65)	0.69 (0.32, 1.49)	0.89 (0.44, 1.79)	0.78	
HPV18						
Negative	1.00 (Ref)	0.57 (0.37, 0.85)	0.45 (0.29, 0.69)	0.53 (0.35, 0.81)	0.001	0.53
Positive	1.00 (Ref)	0.65 (0.14, 2.51)	0.55 (0.16, 1.87)	0.33 (0.10, 1.12)	0.07	
HPV52						
Negative	1.00 (Ref)	0.56 (0.35, 0.88)	0.47 (0.30, 0.75)	0.59 (0.37, 0.92)	0.01	0.16
Positive	1.00 (Ref)	0.59 (0.25, 1.29)	0.42 (0.18, 0.99)	0.31 (0.14, 0.72)	0.004	
HPV58						
Negative	1.00 (Ref)	0.53 (0.36, 0.81)	0.43 (0.28, 0.65)	0.51 (0.33, 0.78)	0.001	0.76
Positive	1.00 (Ref)	1.05 (0.18, 3.21)	0.82 (0.24, 2.8)	0.60 (0.20, 1.81)	0.35	
Others						
Negative	1.00 (Ref)	0.88 (0.33, 1.47)	0.74 (0.44, 1.26)	0.64 (0.39, 1.03)	0.05	0.09
Positive	1.00 (Ref)	0.31 (0.29, 0.58)	0.22 (0.12, 0.42)	0.38 (0.19, 0.76)	0.001	
Multiple infection						
No	1.00 (Ref)	0.64 (0.36, 0.98)	0.49 (0.31, 0.76)	0.54 (0.35, 0.84)	0.002	0.89
Yes	1.00 (Ref)	0.34 (0.19, 0.98)	0.26 (0.09, 0.77)	0.31 (0.11, 0.86)	0.03	

Abbreviations: CI, confidence interval; HPV, human papillomavirus; OR, odds ratio; Ref, reference.

a Logistic regression models with adjustment for age, HPV16, 18, 52, 58, and the other high‐risk HPV types where appropriate.

Interaction analysis by adding a multiplicative interaction term in logistic regression models.

## 4 DISCUSSION

In this case‐control study, we observed the dose‐response relationship of shorter telomere length in cervical exfoliated cells with lower risk of cervical cancer after adjusting HR‐HPV types. The association appeared to be stronger in non‐HPV16 infections than positive infection. HPV16 has stronger ability activate telomerase, which may lead to telomere elongation. For our results, HPV16 was positively correlated with telomere length, but there was no interaction between telomeres and HPV16. Some studies suggested that telomere length might represent a genetic factor for cervical cancer. Telomeres might play a role in cervical cancer susceptibility and interact with specific HPV types in carcinogenesis. In this cross‐sectional study, it was hard to distinguish the genetic and environmental effects on cervical cancer development. The interaction between telomere length and non‐HPV16 types requires further studies. This was the first effort to determine the association between cervical cancer risk and telomere length among HR‐HPV‐positive women. Our results confirmed that telomeres might play an important role in cervical carcinogenesis, and shortened telomere length could be a protective factor.

Although the association between cancer risk and telomere length has been widely investigated for skin cancer, breast cancer, lung cancer, and gastrointestinal tumor,^27^, ^28^ epidemiologic evidence for cervical cancer remains scarce. An early study reported that cervical intraepithelial neoplasia (CIN) had shorter telomeres than corresponding normal epithelia, indicating the active role of telomeres in cervical carcinogenesis.^29^ The sample size was very small in that study (n = 20 for CIN) and HPV infection was not determined, which could result in bias. The current study was based on a cytology‐based screening program, thus cervical exfoliated cell samples were collected from each participant. After HPV detection and genotyping, we determined RTL in cervical exfoliated cells from 591 cervical cancer patients and 373 cancer‐free controls, all of whom were HR‐HPV positive. Our findings indicated that shorter telomere length was a protective factor for cervical cancer, which might serve as a biomarker to identify higher risk individuals during cervical cancer screening. Prospective studies are necessary to validate our findings and confirm the potential of telomere length as a novel biomarker.

Short telomeres may perform as a tumor suppressive role by limiting the replicative potential of cells.^20^ With every cell division, telomeres lose up to 300 bp of DNA and critically short telomeres block further cell division through checkpoint signaling pathways. Telomerase elongates telomeres by adding telomere repeats during S phase and into M phase. In many cancers, telomerase is reactivated to maintain telomere length, and telomerase is pursued as the drug target for cancer treatment.^30^ Several studies reported increased telomerase activity in high‐grade cervical lesions compared to low‐grade or normal cytology.^31^, ^32^, ^33^ Infections with HPV 16 and 18 positively correlated with increased telomerase activity.^34^ HPV16 E6 with E6AP could induce the expression of the catalytic subunit of the telomerase, hTERT transcription and telomerase activity, thereby maintaining telomere length and cell proliferation.^35^ In addition, HPV16 E7 also contributed to the telomerase induction and maintenance of its activity.^14^ These evidences explained our finding that HPV16 was positively correlated with telomere length in cervical exfoliated cells. Of note, we found that the protective effect of short telomere was restricted to non‐HPV16 infections. It is possible that different types of HR‐HPV may differentially affect telomerase activity and telomere length. Potential interactions between different HR‐HPV and telomere length warrant further investigation in experimental studies.

This study had several strengths, including large cohort and HPV genotyping, which allowed to discreetly calculate the dose‐response relationship between telomere length and cervical cancer risk. However, our study was case‐control design, which limited the ability to make causal inference. The role of longer telomere remains inconclusive for cervical cancer. Additionally, our results were based on Chinese population, and further studies are warranted for different populations.

## 5 CONCLUSION

Our study provided novel epidemiologic evidence on the relationship between telomere and cervical cancer. Shortened telomere might act as a protective factor in cervical carcinogenesis. Further prospective studies with large cohort should be designed to validate our preliminary findings, and evaluate the potential efficacy of telomere length for cervical cancer discrimination.

## CONFLICT OF INTEREST

The authors declare that they have no conflict of interest.
